# Di-μ-bromido-bis­[bromido(4,7-diphenyl-1,10-phenanthroline-κ^2^ *N*,*N*′)cadmium]

**DOI:** 10.1107/S1600536812045928

**Published:** 2012-11-10

**Authors:** Sadif A. Shirvan, Sara Haydari Dezfuli, Fereydoon Khazali, Manouchehr Aghajeri, Ali Borsalani

**Affiliations:** a Department of Chemistry, Omidieh Branch, Islamic Azad University, Omidieh, Iran; b Department of Petroleum Engineering, Omidieh Branch, Islamic Azad University, Omidieh, Iran

## Abstract

The title compound, [Cd_2_Br_4_(C_24_H_16_N_2_)_2_], consists of a centrosymmetric dimeric unit in which two Br atoms bridge two Cd^II^ atoms, forming a four-membered ring. A terminal Br atom and a bidentate chelating 4,7-diphenyl-1,10-phenanthroline ligand complete a square-pyramidal geometry for the Cd^II^ atom. In the crystal, C—H⋯Br hydrogen bonds and π–π contacts between the pyridine and phenyl rings [centroid–centroid distances = 3.704 (4) and 3.715 (4) Å] lead to a three-dimensional supra­molecular structure.

## Related literature

For related structures, see: Abedi *et al.* (2012 ▶); Ahmadi *et al.* (2008 ▶); Alizadeh *et al.* (2010 ▶); Chesnut *et al.* (2001 ▶); Gaballa *et al.* (2003 ▶); Yousefi *et al.* (2008 ▶).
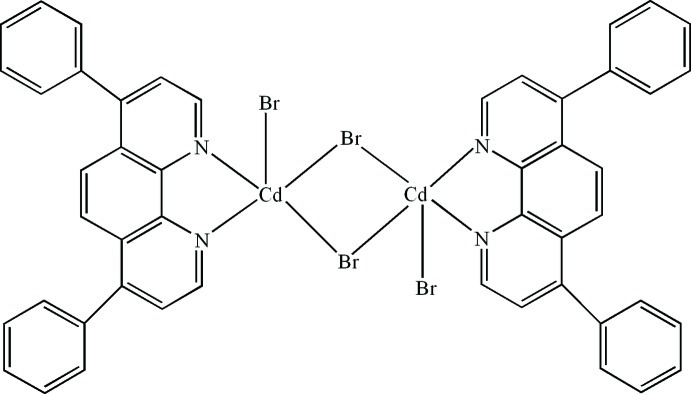


## Experimental

### 

#### Crystal data

[Cd_2_Br_4_(C_24_H_16_N_2_)_2_]
*M* *_r_* = 1209.20
Monoclinic, 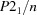

*a* = 10.1704 (4) Å
*b* = 12.4702 (5) Å
*c* = 17.3444 (7) Å
β = 103.187 (3)°
*V* = 2141.73 (15) Å^3^
*Z* = 2
Mo *K*α radiation
μ = 4.76 mm^−1^
*T* = 120 K
0.25 × 0.18 × 0.15 mm

#### Data collection

Bruker APEXII CCD diffractometer
Absorption correction: multi-scan (*SADABS*; Bruker, 2001 ▶) *T*_min_ = 0.385, *T*_max_ = 0.502
12072 measured reflections
4200 independent reflections
3248 reflections with *I* > 2σ(*I*)
*R*_int_ = 0.090

#### Refinement

*R*[*F* ^2^ > 2σ(*F* ^2^)] = 0.052
*wR*(*F* ^2^) = 0.129
*S* = 1.05
4200 reflections
262 parameters
H-atom parameters constrained
Δρ_max_ = 1.15 e Å^−3^
Δρ_min_ = −1.05 e Å^−3^

### 

Data collection: *APEX2* (Bruker, 2007 ▶); cell refinement: *SAINT* (Bruker, 2007 ▶); data reduction: *SAINT*; program(s) used to solve structure: *SHELXS97* (Sheldrick, 2008 ▶); program(s) used to refine structure: *SHELXL97* (Sheldrick, 2008 ▶); molecular graphics: *ORTEP-3* (Farrugia, 2012 ▶) and *Mercury* (Macrae *et al.*, 2006 ▶); software used to prepare material for publication: *SHELXL97*.

## Supplementary Material

Crystal structure: contains datablock(s) I, global. DOI: 10.1107/S1600536812045928/hy2601sup1.cif

Structure factors: contains datablock(s) I. DOI: 10.1107/S1600536812045928/hy2601Isup2.hkl

Additional supplementary materials:  crystallographic information; 3D view; checkCIF report

## Figures and Tables

**Table 1:** Selected bond lengths (Å)

Cd1—N1	2.336 (6)
Cd1—N2	2.349 (6)
Cd1—Br1	2.5537 (9)
Cd1—Br2	2.6653 (8)
Cd1—Br2^i^	2.7518 (9)

Symmetry code: (i) 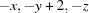.

**Table 2:** Hydrogen-bond geometry (Å, °)

*D*—H⋯*A*	*D*—H	H⋯*A*	*D*⋯*A*	*D*—H⋯*A*
C1—H1⋯Br2^i^	0.93	2.90	3.554 (7)	129
C21—H21⋯Br1^ii^	0.93	2.79	3.582 (8)	144

Symmetry codes: (i) 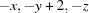; (ii) 
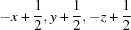
.

**Fig. 1.**
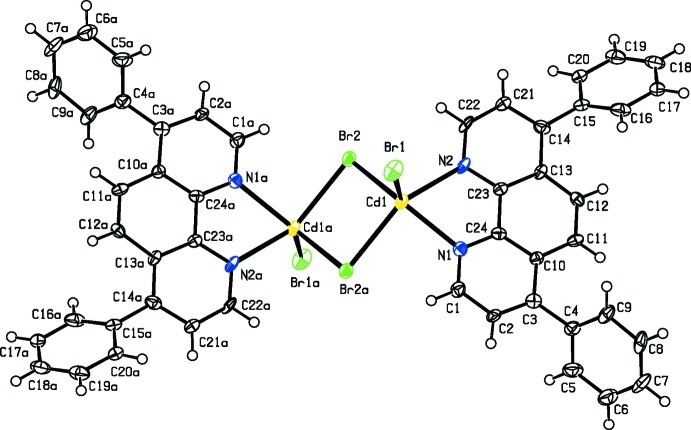
The molecular structure of the title compound. Displacement ellipsoids are drawn at the 50% probability level.

**Fig. 2.**
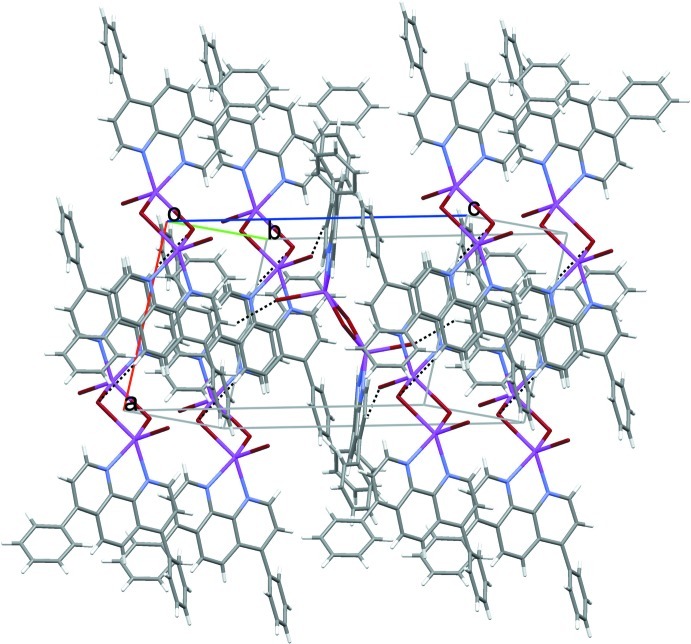
Crystal packing diagram for the title compound. Hydrogen bonds are shown as dashed lines.

## supplementary crystallographic information

### Comment

4,7-Diphenyl-1,10-phenanthroline (Ph_2_phen) is a good bidentate ligand, and numerous complexes with Ph_2_phen have been prepared, such as that of mercury (Alizadeh *et al.*, 2010; Yousefi *et al.*, 2008), gold (Ahmadi *et al.*, 2008), indium (Abedi *et al.*, 2012), copper (Chesnut *et al.*, 2001) and platinum (Gaballa *et al.*, 2003). Here, we report the synthesis and structure of the title compound.

The structure of the title compound (Fig. 1) consists of a centrosymmetric dimeric unit, [Cd_2_Br_4_(Ph_2_phen)_2_], in which two Br atoms bridge two Cd^II^ atoms, forming a four-membered ring; a terminal Br atom and a bidentate chelating Ph_2_phen complete a five coordination (Table 1). In the crystal structure, intermolecular C—H···Br hydrogen bonds (Table 2) and π–π contacts (Fig. 2) between the pyridine and phenyl rings, *Cg*3···*Cg*4^i^ and *Cg*6···*Cg*6^i^ [symmetry code: (i) 1-x, 2-y, -z, *Cg*3, *Cg*4 and *Cg*6 are the centroids of the rings N1/C1–C3/C10/C24, N2/C13–C14/C21–C23 and C10–C13/C23–C24, respectively], with centroid–centroid distances of 3.704 (4) and 3.715 (4) Å, stabilize the structure.

### Experimental

For the preparation of the title compound, a solution of Ph_2_phen (0.44 g, 1.33 mmol) in methanol (10 ml) was added to a solution of CdBr_2_.4H_2_O (0.46 g, 1.33 mmol) in methanol (10 ml) at room temperature. Crystals suitable for X-ray diffraction experiment were obtained by methanol diffusion into a colorless solution in DMSO after one week (yield: 0.62 g, 77.1%).

### Refinement

All H atoms were positioned geometrically and refined as riding atoms, with C—H = 0.93 Å and *U*_iso_(H) = 1.2*U*_eq_(C).

### Crystal data

**Table:**

[Cd_2_Br_4_(C_24_H_16_N_2_)_2_]	*F*(000) = 1168
*M**_r_* = 1209.20	*D*_x_ = 1.875 Mg m^−^^3^
Monoclinic, *P*2_1_/*n*	Mo *K*α radiation, λ = 0.71073 Å
Hall symbol: -P 2yn	Cell parameters from 12072 reflections
*a* = 10.1704 (4) Å	θ = 2.4–26.0°
*b* = 12.4702 (5) Å	µ = 4.76 mm^−^^1^
*c* = 17.3444 (7) Å	*T* = 120 K
β = 103.187 (3)°	Prism, colorless
*V* = 2141.73 (15) Å^3^	0.25 × 0.18 × 0.15 mm
*Z* = 2	

### Data collection

**Table:**

Bruker APEXII CCD diffractometer	4200 independent reflections
Radiation source: fine-focus sealed tube	3248 reflections with *I* > 2σ(*I*)
Graphite monochromator	*R*_int_ = 0.090
φ and ω scans	θ_max_ = 26.0°, θ_min_ = 2.4°
Absorption correction: multi-scan (*SADABS*; Bruker, 2001)	*h* = −12→9
*T*_min_ = 0.385, *T*_max_ = 0.502	*k* = −15→15
12072 measured reflections	*l* = −21→21

### Refinement

**Table:**

Refinement on *F*^2^	Primary atom site location: structure-invariant direct methods
Least-squares matrix: full	Secondary atom site location: difference Fourier map
*R*[*F*^2^ > 2σ(*F*^2^)] = 0.052	Hydrogen site location: inferred from neighbouring sites
*wR*(*F*^2^) = 0.129	H-atom parameters constrained
*S* = 1.05	*w* = 1/[σ^2^(*F*_o_^2^) + (0.0692*P*)^2^] where *P* = (*F*_o_^2^ + 2*F*_c_^2^)/3
4200 reflections	(Δ/σ)_max_ = 0.005
262 parameters	Δρ_max_ = 1.15 e Å^−^^3^
0 restraints	Δρ_min_ = −1.05 e Å^−^^3^

### Special details

**Table:**

Geometry. All e.s.d.'s (except the e.s.d. in the dihedral angle between two l.s. planes) are estimated using the full covariance matrix. The cell e.s.d.'s are taken into account individually in the estimation of e.s.d.'s in distances, angles and torsion angles; correlations between e.s.d.'s in cell parameters are only used when they are defined by crystal symmetry. An approximate (isotropic) treatment of cell e.s.d.'s is used for estimating e.s.d.'s involving l.s. planes.
Refinement. Refinement of *F*^2^ against ALL reflections. The weighted *R*-factor *wR* and goodness of fit *S* are based on *F*^2^, conventional *R*-factors *R* are based on *F*, with *F* set to zero for negative *F*^2^. The threshold expression of *F*^2^ > σ(*F*^2^) is used only for calculating *R*-factors(gt) *etc*. and is not relevant to the choice of reflections for refinement. *R*-factors based on *F*^2^ are statistically about twice as large as those based on *F*, and *R*- factors based on ALL data will be even larger.

### Fractional atomic coordinates and isotropic or equivalent isotropic displacement parameters (Å^2^)

**Table:**

	*x*	*y*	*z*	*U*_iso_*/*U*_eq_	
C1	0.3033 (7)	0.7552 (6)	0.0038 (4)	0.0220 (15)	
H1	0.2143	0.7327	−0.0137	0.026*	
C2	0.4054 (7)	0.6893 (6)	−0.0089 (4)	0.0212 (15)	
H2	0.3842	0.6233	−0.0336	0.025*	
C3	0.5378 (7)	0.7205 (5)	0.0147 (4)	0.0198 (14)	
C4	0.6478 (8)	0.6468 (5)	0.0047 (5)	0.0230 (15)	
C5	0.6445 (9)	0.6043 (6)	−0.0702 (5)	0.0342 (19)	
H5	0.5770	0.6250	−0.1134	0.041*	
C6	0.7429 (9)	0.5306 (7)	−0.0800 (6)	0.044 (2)	
H6	0.7435	0.5048	−0.1302	0.052*	
C7	0.8396 (9)	0.4960 (6)	−0.0150 (6)	0.039 (2)	
H7	0.9039	0.4457	−0.0212	0.047*	
C8	0.8401 (8)	0.5363 (7)	0.0590 (6)	0.0329 (18)	
H8	0.9037	0.5117	0.1028	0.040*	
C9	0.7462 (8)	0.6139 (6)	0.0690 (5)	0.0281 (17)	
H9	0.7500	0.6432	0.1187	0.034*	
C10	0.5663 (7)	0.8236 (5)	0.0511 (4)	0.0171 (14)	
C11	0.6978 (8)	0.8691 (6)	0.0725 (4)	0.0206 (15)	
H11	0.7700	0.8317	0.0608	0.025*	
C12	0.7213 (7)	0.9657 (6)	0.1095 (4)	0.0187 (14)	
H12	0.8079	0.9947	0.1206	0.022*	
C13	0.6142 (7)	1.0227 (5)	0.1312 (4)	0.0163 (13)	
C14	0.6353 (8)	1.1181 (6)	0.1777 (4)	0.0226 (15)	
C15	0.7704 (7)	1.1668 (5)	0.2081 (4)	0.0195 (14)	
C16	0.8805 (9)	1.1070 (6)	0.2449 (4)	0.0303 (18)	
H16	0.8708	1.0338	0.2523	0.036*	
C17	1.0068 (8)	1.1553 (7)	0.2711 (5)	0.0318 (19)	
H17	1.0812	1.1140	0.2948	0.038*	
C18	1.0212 (9)	1.2650 (7)	0.2618 (5)	0.037 (2)	
H18	1.1052	1.2975	0.2789	0.045*	
C19	0.9106 (9)	1.3248 (7)	0.2272 (4)	0.0301 (18)	
H19	0.9203	1.3982	0.2204	0.036*	
C20	0.7865 (7)	1.2787 (6)	0.2025 (4)	0.0229 (15)	
H20	0.7118	1.3214	0.1817	0.027*	
C21	0.5205 (8)	1.1675 (6)	0.1945 (5)	0.0261 (16)	
H21	0.5298	1.2292	0.2254	0.031*	
C22	0.3948 (7)	1.1245 (6)	0.1654 (4)	0.0236 (16)	
H22	0.3213	1.1601	0.1770	0.028*	
C23	0.4797 (7)	0.9847 (5)	0.1065 (4)	0.0159 (13)	
C24	0.4563 (7)	0.8838 (5)	0.0639 (4)	0.0172 (14)	
N1	0.3274 (6)	0.8502 (5)	0.0403 (3)	0.0190 (12)	
N2	0.3701 (6)	1.0356 (5)	0.1218 (3)	0.0197 (12)	
Cd1	0.15894 (5)	0.95338 (4)	0.07604 (3)	0.01781 (15)	
Br1	0.10996 (9)	0.87751 (7)	0.20336 (5)	0.0309 (2)	
Br2	0.03037 (7)	1.14002 (5)	0.04404 (4)	0.02161 (18)	

### Atomic displacement parameters (Å^2^)

**Table:**

	*U*^11^	*U*^22^	*U*^33^	*U*^12^	*U*^13^	*U*^23^
C1	0.020 (4)	0.023 (4)	0.026 (4)	−0.002 (3)	0.011 (3)	0.000 (3)
C2	0.019 (3)	0.024 (4)	0.024 (3)	−0.001 (3)	0.012 (3)	−0.008 (3)
C3	0.025 (4)	0.016 (3)	0.018 (3)	0.000 (3)	0.005 (3)	0.003 (3)
C4	0.024 (4)	0.015 (3)	0.032 (4)	−0.004 (3)	0.011 (3)	−0.004 (3)
C5	0.040 (5)	0.024 (4)	0.038 (5)	−0.004 (4)	0.010 (4)	−0.012 (4)
C6	0.038 (5)	0.033 (5)	0.062 (6)	−0.001 (4)	0.017 (4)	−0.022 (4)
C7	0.031 (4)	0.018 (4)	0.079 (7)	−0.002 (3)	0.037 (5)	−0.012 (4)
C8	0.016 (3)	0.031 (4)	0.055 (5)	0.001 (3)	0.017 (3)	0.008 (4)
C9	0.026 (4)	0.025 (4)	0.040 (4)	0.002 (3)	0.023 (4)	0.002 (3)
C10	0.019 (3)	0.016 (3)	0.016 (3)	0.002 (3)	0.004 (3)	0.001 (3)
C11	0.027 (4)	0.022 (3)	0.015 (3)	0.002 (3)	0.010 (3)	−0.003 (3)
C12	0.018 (3)	0.021 (3)	0.016 (3)	−0.005 (3)	0.003 (3)	0.001 (3)
C13	0.017 (3)	0.021 (3)	0.015 (3)	0.002 (3)	0.012 (3)	0.004 (3)
C14	0.027 (4)	0.017 (3)	0.022 (3)	−0.002 (3)	0.001 (3)	−0.002 (3)
C15	0.026 (4)	0.017 (3)	0.017 (3)	0.001 (3)	0.007 (3)	−0.004 (3)
C16	0.044 (5)	0.026 (4)	0.018 (4)	0.002 (4)	0.002 (3)	−0.005 (3)
C17	0.024 (4)	0.047 (5)	0.023 (4)	0.006 (4)	0.002 (3)	−0.016 (4)
C18	0.034 (5)	0.046 (5)	0.034 (4)	−0.016 (4)	0.014 (4)	−0.024 (4)
C19	0.038 (5)	0.032 (4)	0.023 (4)	−0.010 (4)	0.011 (3)	−0.008 (3)
C20	0.023 (4)	0.025 (4)	0.022 (3)	0.002 (3)	0.008 (3)	−0.006 (3)
C21	0.024 (4)	0.021 (4)	0.033 (4)	0.006 (3)	0.006 (3)	−0.008 (3)
C22	0.020 (4)	0.026 (4)	0.031 (4)	0.006 (3)	0.020 (3)	−0.001 (3)
C23	0.020 (3)	0.016 (3)	0.013 (3)	0.001 (3)	0.007 (3)	0.002 (2)
C24	0.023 (4)	0.014 (3)	0.013 (3)	0.001 (3)	0.002 (3)	0.001 (3)
N1	0.018 (3)	0.022 (3)	0.019 (3)	−0.002 (2)	0.007 (2)	0.001 (2)
N2	0.019 (3)	0.023 (3)	0.023 (3)	0.005 (3)	0.016 (2)	0.003 (3)
Cd1	0.0164 (3)	0.0199 (3)	0.0193 (2)	0.0026 (2)	0.00858 (18)	0.0045 (2)
Br1	0.0369 (4)	0.0325 (4)	0.0291 (4)	0.0053 (3)	0.0195 (3)	0.0146 (3)
Br2	0.0191 (3)	0.0189 (3)	0.0267 (4)	0.0035 (3)	0.0050 (3)	0.0001 (3)

### Geometric parameters (Å, º)

**Table:**

C1—N1	1.339 (9)	C14—C21	1.409 (11)
C1—C2	1.381 (10)	C14—C15	1.484 (10)
C1—H1	0.9300	C15—C16	1.375 (11)
C2—C3	1.372 (10)	C15—C20	1.411 (10)
C2—H2	0.9300	C16—C17	1.398 (12)
C3—C10	1.432 (9)	C16—H16	0.9300
C3—C4	1.489 (10)	C17—C18	1.389 (13)
C4—C9	1.379 (11)	C17—H17	0.9300
C4—C5	1.396 (11)	C18—C19	1.369 (13)
C5—C6	1.398 (12)	C18—H18	0.9300
C5—H5	0.9300	C19—C20	1.365 (11)
C6—C7	1.385 (14)	C19—H19	0.9300
C6—H6	0.9300	C20—H20	0.9300
C7—C8	1.377 (13)	C21—C22	1.371 (11)
C7—H7	0.9300	C21—H21	0.9300
C8—C9	1.398 (11)	C22—N2	1.333 (9)
C8—H8	0.9300	C22—H22	0.9300
C9—H9	0.9300	C23—N2	1.361 (9)
C10—C24	1.406 (10)	C23—C24	1.452 (9)
C10—C11	1.423 (10)	C24—N1	1.349 (9)
C11—C12	1.361 (9)	Cd1—N1	2.336 (6)
C11—H11	0.9300	Cd1—N2	2.349 (6)
C12—C13	1.421 (10)	Cd1—Br1	2.5537 (9)
C12—H12	0.9300	Cd1—Br2	2.6653 (8)
C13—C23	1.418 (9)	Cd1—Br2^i^	2.7518 (9)
C13—C14	1.426 (9)		
			
N1—C1—C2	122.6 (7)	C15—C16—C17	120.5 (8)
N1—C1—H1	118.7	C15—C16—H16	119.8
C2—C1—H1	118.7	C17—C16—H16	119.8
C3—C2—C1	120.4 (7)	C18—C17—C16	120.1 (8)
C3—C2—H2	119.8	C18—C17—H17	120.0
C1—C2—H2	119.8	C16—C17—H17	120.0
C2—C3—C10	118.2 (7)	C19—C18—C17	119.3 (8)
C2—C3—C4	120.2 (6)	C19—C18—H18	120.4
C10—C3—C4	121.6 (6)	C17—C18—H18	120.4
C9—C4—C5	119.9 (7)	C20—C19—C18	121.1 (8)
C9—C4—C3	121.0 (7)	C20—C19—H19	119.4
C5—C4—C3	118.9 (7)	C18—C19—H19	119.4
C4—C5—C6	119.8 (8)	C19—C20—C15	120.6 (7)
C4—C5—H5	120.1	C19—C20—H20	119.7
C6—C5—H5	120.1	C15—C20—H20	119.7
C7—C6—C5	120.0 (9)	C22—C21—C14	120.0 (7)
C7—C6—H6	120.0	C22—C21—H21	120.0
C5—C6—H6	120.0	C14—C21—H21	120.0
C8—C7—C6	119.8 (8)	N2—C22—C21	125.0 (7)
C8—C7—H7	120.1	N2—C22—H22	117.5
C6—C7—H7	120.1	C21—C22—H22	117.5
C7—C8—C9	120.7 (8)	N2—C23—C13	124.2 (6)
C7—C8—H8	119.7	N2—C23—C24	117.2 (6)
C9—C8—H8	119.7	C13—C23—C24	118.6 (6)
C4—C9—C8	119.7 (8)	N1—C24—C10	122.7 (6)
C4—C9—H9	120.1	N1—C24—C23	117.3 (6)
C8—C9—H9	120.1	C10—C24—C23	120.0 (6)
C24—C10—C11	118.7 (6)	C1—N1—C24	118.6 (6)
C24—C10—C3	117.4 (6)	C1—N1—Cd1	123.1 (5)
C11—C10—C3	123.8 (7)	C24—N1—Cd1	118.0 (4)
C12—C11—C10	122.0 (7)	C22—N2—C23	116.2 (6)
C12—C11—H11	119.0	C22—N2—Cd1	126.5 (5)
C10—C11—H11	119.0	C23—N2—Cd1	117.2 (4)
C11—C12—C13	120.4 (6)	N1—Cd1—N2	70.2 (2)
C11—C12—H12	119.8	N1—Cd1—Br1	108.95 (14)
C13—C12—H12	119.8	N2—Cd1—Br1	102.32 (14)
C23—C13—C12	119.8 (6)	N1—Cd1—Br2	141.29 (14)
C23—C13—C14	117.4 (6)	N2—Cd1—Br2	93.23 (14)
C12—C13—C14	122.8 (6)	Br1—Cd1—Br2	108.68 (3)
C21—C14—C13	117.2 (7)	N1—Cd1—Br2^i^	89.71 (14)
C21—C14—C15	119.5 (6)	N2—Cd1—Br2^i^	150.28 (14)
C13—C14—C15	123.3 (7)	Br1—Cd1—Br2^i^	104.89 (3)
C16—C15—C20	118.3 (7)	Br2—Cd1—Br2^i^	89.24 (3)
C16—C15—C14	122.2 (7)	Cd1—Br2—Cd1^i^	90.76 (3)
C20—C15—C14	119.5 (7)		
			
N1—C1—C2—C3	−1.2 (11)	C14—C13—C23—N2	2.6 (10)
C1—C2—C3—C10	−1.3 (10)	C12—C13—C23—C24	4.2 (9)
C1—C2—C3—C4	176.8 (7)	C14—C13—C23—C24	−175.7 (6)
C2—C3—C4—C9	−120.8 (8)	C11—C10—C24—N1	175.1 (6)
C10—C3—C4—C9	57.2 (10)	C3—C10—C24—N1	−2.9 (10)
C2—C3—C4—C5	54.2 (10)	C11—C10—C24—C23	−7.1 (9)
C10—C3—C4—C5	−127.8 (8)	C3—C10—C24—C23	174.8 (6)
C9—C4—C5—C6	−1.6 (12)	N2—C23—C24—N1	2.3 (9)
C3—C4—C5—C6	−176.7 (7)	C13—C23—C24—N1	−179.2 (6)
C4—C5—C6—C7	3.1 (13)	N2—C23—C24—C10	−175.6 (6)
C5—C6—C7—C8	−1.5 (13)	C13—C23—C24—C10	2.9 (9)
C6—C7—C8—C9	−1.6 (12)	C2—C1—N1—C24	1.6 (10)
C5—C4—C9—C8	−1.5 (11)	C2—C1—N1—Cd1	−172.4 (5)
C3—C4—C9—C8	173.5 (7)	C10—C24—N1—C1	0.6 (10)
C7—C8—C9—C4	3.1 (11)	C23—C24—N1—C1	−177.3 (6)
C2—C3—C10—C24	3.2 (9)	C10—C24—N1—Cd1	174.8 (5)
C4—C3—C10—C24	−174.8 (6)	C23—C24—N1—Cd1	−3.0 (8)
C2—C3—C10—C11	−174.7 (7)	C21—C22—N2—C23	0.7 (10)
C4—C3—C10—C11	7.2 (10)	C21—C22—N2—Cd1	176.5 (6)
C24—C10—C11—C12	4.4 (10)	C13—C23—N2—C22	−2.6 (9)
C3—C10—C11—C12	−177.7 (6)	C24—C23—N2—C22	175.7 (6)
C10—C11—C12—C13	2.7 (10)	C13—C23—N2—Cd1	−178.8 (5)
C11—C12—C13—C23	−7.1 (9)	C24—C23—N2—Cd1	−0.5 (7)
C11—C12—C13—C14	172.8 (6)	C1—N1—Cd1—N2	176.0 (6)
C23—C13—C14—C21	−0.7 (9)	C24—N1—Cd1—N2	2.0 (5)
C12—C13—C14—C21	179.4 (7)	C1—N1—Cd1—Br1	79.3 (5)
C23—C13—C14—C15	179.6 (6)	C24—N1—Cd1—Br1	−94.7 (5)
C12—C13—C14—C15	−0.3 (10)	C1—N1—Cd1—Br2	−114.7 (5)
C21—C14—C15—C16	133.3 (8)	C24—N1—Cd1—Br2	71.2 (5)
C13—C14—C15—C16	−47.1 (11)	C1—N1—Cd1—Br2^i^	−26.3 (5)
C21—C14—C15—C20	−44.0 (10)	C24—N1—Cd1—Br2^i^	159.7 (5)
C13—C14—C15—C20	135.7 (7)	C22—N2—Cd1—N1	−176.5 (6)
C20—C15—C16—C17	−4.2 (11)	C23—N2—Cd1—N1	−0.7 (4)
C14—C15—C16—C17	178.5 (7)	C22—N2—Cd1—Br1	−70.6 (6)
C15—C16—C17—C18	1.5 (12)	C23—N2—Cd1—Br1	105.2 (4)
C16—C17—C18—C19	0.3 (12)	C22—N2—Cd1—Br2	39.3 (6)
C17—C18—C19—C20	0.6 (12)	C23—N2—Cd1—Br2	−144.9 (4)
C18—C19—C20—C15	−3.4 (11)	C22—N2—Cd1—Br2^i^	133.5 (5)
C16—C15—C20—C19	5.2 (11)	C23—N2—Cd1—Br2^i^	−50.7 (6)
C14—C15—C20—C19	−177.5 (7)	N1—Cd1—Br2—Cd1^i^	88.6 (2)
C13—C14—C21—C22	−1.0 (11)	N2—Cd1—Br2—Cd1^i^	150.36 (14)
C15—C14—C21—C22	178.7 (7)	Br1—Cd1—Br2—Cd1^i^	−105.47 (3)
C14—C21—C22—N2	1.1 (12)	Br2^i^—Cd1—Br2—Cd1^i^	0.0
C12—C13—C23—N2	−177.5 (6)		

Symmetry code: (i) −*x*, −*y*+2, −*z*.

### Hydrogen-bond geometry (Å, º)

**Table:**

*D*—H···*A*	*D*—H	H···*A*	*D*···*A*	*D*—H···*A*
C1—H1···Br2^i^	0.93	2.90	3.554 (7)	129
C21—H21···Br1^ii^	0.93	2.79	3.582 (8)	144

Symmetry codes: (i) −*x*, −*y*+2, −*z*; (ii) −*x*+1/2, *y*+1/2, −*z*+1/2.
